# Diversity of Macrophages in Lung Homeostasis and Diseases

**DOI:** 10.3389/fimmu.2021.753940

**Published:** 2021-09-24

**Authors:** Fei Hou, Kun Xiao, Li Tang, Lixin Xie

**Affiliations:** ^1^ College of Pulmonary and Critical Care Medicine, Chinese PLA General Hospital, Beijing, China; ^2^ Medical School of Chinese PLA, Beijing, China; ^3^ State Key Laboratory of Proteomics, Beijing Proteome Research Center, National Center for Protein Sciences·Beijing, Beijing Institute of Lifeomics, Beijing, China

**Keywords:** macrophage, inflammation, infection, fibrosis, COVID-19

## Abstract

Lung macrophages play important roles in the maintenance of homeostasis, pathogen clearance and immune regulation. The different types of pulmonary macrophages and their roles in lung diseases have attracted attention in recent years. Alveolar macrophages (AMs), including tissue-resident alveolar macrophages (TR-AMs) and monocyte-derived alveolar macrophages (Mo-AMs), as well as interstitial macrophages (IMs) are the major macrophage populations in the lung and have unique characteristics in both steady-state conditions and disease states. The different characteristics of these three types of macrophages determine the different roles they play in the development of disease. Therefore, it is important to fully understand the similarities and differences among these three types of macrophages for the study of lung diseases. In this review, we will discuss the physiological characteristics and unique functions of these three types of macrophages in acute and chronic lung diseases. We will also discuss possible methods to target macrophages in lung diseases.

## Introduction

In recent years, our understanding of lung immune cell heterogeneity has advanced significantly with the emergence of new technologies, such as single-cell RNA sequencing (scRNA-seq). The COVID-19 pandemic also made us painfully aware of the impact respiratory viral infections can have and sparked interest in the cellular and molecular mechanisms of lung immunity. Lung macrophages, including AMs and IMs, are important innate immune cells involved in the normal physiological functions of lung tissue and some acute and chronic diseases, such as infections and fibrosis (1–3). The microenvironment of the alveoli is different from that of other sites, making TR-AMs different from IMs and other tissue macrophages. Due to their different origins, AMs can be subdivided into TR-AMs and Mo-AMs. Unique growth factors and receptors in the steady state restrict the plasticity of TR-AMs, rendering them hyporesponsive to inhaled particles and dust (4). Following insults, newly recruited Mo-AMs move to the alveoli, join the AM pool and develop their own features, while the monocyte lineage is more plastic than mature AMs (5, 6). Early inflammatory Mo-AMs may be associated with immune disorders such as cytokine storms in some infectious diseases, and late profibrotic Mo-AMs are associated with lung fibrosis (6–9). By summarizing recent studies of lung macrophages, we concluded that TR-AMs function as sentinels that maintain immune balance while the characteristics and functions of Mo-AMs are mainly dependent on the lung microenvironment. Homeostatic maintenance, immune surveillance, phagocytosis and inflammatory resolution may be performed by these three types of macrophages together or separately and these cells may communicate to maintain the immune balance (10–13). Defining the exact role of different lung macrophages in pathology and diseases may help to identify the cause and find appropriate therapeutic strategies.

## Macrophage Subsets in the Lung

**Figure 1:**
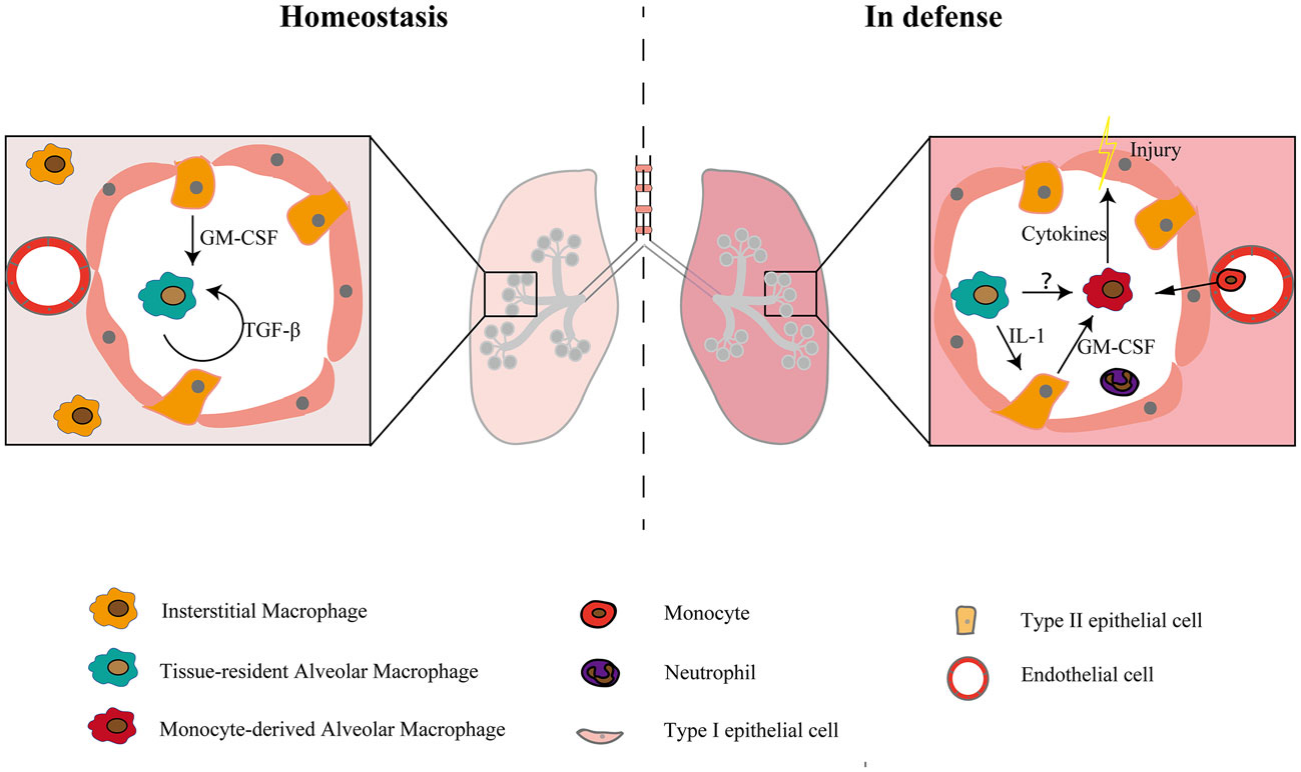
Macrophage subsets in the steady state and in defense. There are two populations of macrophages in the physiological state and three populations of macrophages in the injury and inflammatory states. In the steady-state condition, the maturation and self-maintenance of TR-AMs rely on GM-CSF and TGF-β. When injury occurs, monocytes recruit to the alveolar lumen and develop into macrophages, constituting a second group of alveolar macrophages and causing tissue damage by releasing cytokines. TR-AMs can indirectly affect the functions of Mo-AMs and other myeloid cells by inducing epithelial cells to release GM-CSF (12). Whether there is a direct interaction between TR-AMs and Mo-AMs is still unclear.

The characteristics and functions of different types of macrophages in various organs are gradually being discovered (14–18). Location and origin are the two main factors that determine the characteristics of lung macrophages (19). The lung contains the following two different macrophage subsets based on anatomical position and function: AMs and IMs. AMs exist in the alveolar cavity, while IMs exist in the interstitium (20, 21). When infection or injury occurs, monocytes enter the alveolar cavity and develop into Mo-AMs (**Figure 1**) and they differ substantially from TR-AMs in both phenotypic and metabolic characteristics in the first few days (9, 22–24). Thus, AMs contain two distinct subpopulations as follows: TR-AMs and Mo-AMs. TR-AMs seem to have lower responsiveness and limited plasticity, while Mo-AMs are more likely to be remodeled by the microenvironment (5, 6). The different characteristics of these two types of macrophages determine their distinct functions in lung diseases (19, 25, 26). Due to the widespread use of single-cell and tracer technologies, the subpopulations and physiological characteristics of IMs are gradually being elucidated, and their roles in lung diseases such as fibrosis and infections are also beginning to be discovered. IMs can also be divided into TR-IMs and Mo-IMs according to their origins. However, little is known about the differences in the functions and characteristics of these two types of IMs, so the present review will discuss TR-IMs and Mo-IMs together.

## Resident Alveolar Macrophages: Terminally Differentiated Sentinels

### Physiological Characteristics

TR-AMs reside in the alveolar cavity and have important functions in the turnover of pulmonary surfactant and the removal of dead cells from the alveoli (27–29). TR-AMs originate from yolk sac-derived erythromyeloid progenitors and fetal liver monocytes (30–32). Many factors are involved in AM maturation and self-maintenance (32–35), among which GM-CSF and TGF-β are the most important (**Figure 1**). Defective production of GM-CSF may affect the process of AM maturation, thereby rendering mice more susceptible to pathogens (36). Several transcription factors, such as Bhlhe40 and Bhlhe41, also regulate alveolar macrophage self-renewal and identity. Decreased proliferation is observed in Bhlhe40/Bhlhe41-deficient alveolar macrophages (37). AM development, maturation, and regeneration are also regulated by epigenetic factors, such as histone deacetylase 3 (38). Basophils can also regulate AM development by promoting the transition of naive macrophages toward the AM signature (39).

Studies have shown that TR-AMs can maintain self-renewal independent of monocytes at the steady state *via* proliferation (40, 41). However, in some states, such as injury or infection, TR-AMs are depleted, and Mo-AMs help restore the AM pool. These recruited Mo-AMs can acquire a resident AM phenotype a few weeks later, but some transcriptional characteristics are still different because of their monocyte origin (5, 24, 42). It remains unclear why Mo-AMs do not fully acquire the characteristics of TR-AMs in the same environment, and the functional differences between such Mo-AMs and TR-AMs remain unclear. Other studies have demonstrated that AMs can refill their niche independent of blood monocytes even after insult. Hashimoto et al. (40) found that TR-AMs repopulate through local proliferation after nongenotoxic ablation and genotoxic insult. Lai et al. (43) also found that TR-AMs restore their numbers through self-renewal during blood-stage malaria, which was distinct from splenic red pulp macrophages and hepatic Kupffer cells. The different results of these studies may be due to the degree of AM depletion and the severity of inflammation (6). When AMs are not severely depleted, they can be restored through proliferation with minimal contribution from circulating monocytes. In some conditions where AMs are substantially depleted, however, the remaining AMs are too few to repopulate in a short time, resulting in the recruitment of monocytes to help restore their numbers.

Because most experimental mice live in a specific pathogen-free (SPF) environment while humans live in an environment with bacteria and dust, the situation becomes more complicated in regard to human lung macrophages. The AM pool in adults may contain multiple types of macrophages, especially after they experience multiple infections and lung diseases in their early life. The origin and self-renewal capability of human AMs remain unclear due to the lack of reliable markers and limitations in sampling and study methods. Using humanized mice, Evren et al. (44) found that blood CD14^+^ monocytes give rise to CD206^+^ CD169^+^ resident AMs and IMs. Using single-cell technology, Byrne et al. (45) found that macrophages in transplanted lungs are predominantly recipient-derived, implying that AM cannot fully self-renew in the transplanted state. Thus, in addition to self-renewal, resident AMs may be replenished by peripheral monocytes in humans, and TR-AMs and Mo-AMs may exist at the same time (46).

### Infections and Lung Injury

TR-AMs are the first line defense against pathogens. Phagocytosis is an important mechanism by which TR-AMs defend against bacterial infections. Aging, smoking and severe systemic diseases, such as trauma and sepsis, impair the phagocytosis of TR-AMs, thereby reducing their ability to fight pathogens (47–49). Neupane et al. (50) found that TR-AMs move between alveoli to phagocytose inhaled bacteria and TR-AM migration is crucial for bacterial clearance, which is impaired during viral infection, rendering the lung susceptible to secondary bacterial infections. Additional studies need to confirm whether TR-AMs move within the alveolar lumen, but at least one group of AMs is sessile and attached to the alveolar wall (13). It is also unknown whether the TR-AMs move toward the bacteria or whether the bacteria encounter the TR-AMs and are engulfed. Equipped with several pathogen-recognition receptors, TR-AMs are quickly activated and release several cytokines and chemokines after the onset of infections. However, TR-AMs are less inflammatory than monocytes and neutrophils (9). Depleting TR-AMs can reduce cytokines in the early phase but have less impact on subsequent cytokine release, such as IL-6 and TNF-a, but it has less effect on subsequent proinflammatory processes (51, 52). TR-AMs are less plastic, and proinflammatory TR-AMs and anti-inflammatory TR-AMs may exist at the same time and can be distinguished by the expression of CXCL2 (53). Despite their function in the initiation of inflammation, TR-AMs also play an important role in inflammatory recovery and injury repair by clearing apoptotic cells, also called efferocytosis, and releasing resolving mediators (54–56). Apoptotic cells, such as neutrophils, express “eat me” signals, and macrophages recognize these signals through the MeR tyrosine kinase (MeRTK) receptor, integrins, scavenger receptors and complement receptors (54). Efferocytosis reduces NO production and tissue injury through the PPAR-δ and PPAR-γ pathways (57). Various lipid mediators secreted by macrophages during efferocytosis, such as lipoxin, resolvin and protectin, also promote inflammation resolution by the 15-lipoxygenase pathway (58). TR-AMs also promote inflammation resolution and tissue repair by secreting a series of factors, such as TGF-β and IL-10, as well as promoting the secretion of GM-CSF by alveolar epithelial cells in LPS-induced lung injury (11, 60, 61). During pathological states, the process of efferocytosis may be impaired. In acute respiratory distress syndrome (ARDS) mice, apoptotic neutrophil clearance is impaired and can be reversed by activating AMP-activated protein kinase (AMPK) or neutralizing high-mobility group box 1 (HMGB1) (62). Some pathogens, such as *Klebsiella pneumoniae* and *Staphylococcus aureus*, can also increase tissue damage by inhibiting the efferocytosis of AMs (63, 64).

TR-AMs can protect bodies from the damaging effect of bacteria and viruses, such as *Staphylococcus aureus*, *Klebsiella pneumonia* and influenza (65–67). Depleting AMs significantly reduces the survival rate and increases lung injury severity. However, some other pathogens, such as *Pseudomonas aeruginosa* and respiratory syncytial virus (RSV), can induce AM pyroptosis or necroptosis, thereby causing severe inflammation and increasing lung injury severity (68, 69). Some intracellular bacteria, such as *Mycobacterium tuberculosis*, can infect TR-AMs and, thus, mediate the spread of *M. tuberculosis* from the alveoli to the lungs (70). TR-AMs may also be related to nonpathogen-induced lung injuries, such as ventilator-induced lung injury, and a reduction in TR-AMs reduce the severity of ventilator-induced lung injury (71). Therefore, the role of TR-AMs in infections is complex and difficult to define in terms of good or bad, and their role depends on specific pathogens.

### Type II Inflammation

Atopic diseases, such as allergies and asthma, as well as certain infections caused by parasitic helminths, can induce type II immune responses (72). The type II immune response is mainly regulated by T helper 2 (TH2) cells. TH2 cells mainly secrete the IL-4, IL-5 and IL-13 cytokines and stimulate type II immunity (72). Although airway type II immune responses are mainly mediated by cells like eosinophils, mast cells and basophils, lung macrophages can participate in type II inflammation and affect disease progression (73, 74). Unlike many other macrophages, TR-AMs are hyporesponsive to the canonical type 2 cytokine, IL-4, and parasitic worm infection (75). The reason may be due to the unique lung microenvironment that limits the plasticity of TR-AMs and restricts TR-AMs to a “M2”-like phenotype. Removal of TR-AMs from the alveolar niche restores their ability to respond to type 2 cytokine stimulation (75). Despite their hyporesponsiveness to the canonical type 2 cytokine *in vivo*, TR-AMs can suppress asthmatic lung inflammation caused by house dust mites in mice (25, 76). Depleting TR-AMs decreased IL-27 levels and exacerbated type II inflammation caused by IL-13 and house dust mite allergens (77). TR-AMs can alleviate type II inflammation in many ways. Antigen-bearing TR-AMs can promote the development of Foxp3^+^ regulatory T cells, which contribute to airway tolerance and prevent the development of asthmatic lung inflammation (78). Apoptotic cell engulfment in TR-AMs promotes the production of the regulatory T cell-inducing molecule retinoic acid, which impacts the development of allergen-induced asthmatic airway inflammation (76). Intrinsic TGF-β1 in TR-AMs is essential for TR-AM maturation and is also involved in the control of allergic reactions (59). TGF-β1-deficient AMs expressed enhanced levels of monocyte-attractant chemokines and displayed augmented type II inflammation to house dust mite (59). Lung macrophage mannose receptor (MRC1/CD206) also have functions in protection against allergen-induced lung inflammation and *Mrc*1-/- mice had an exacerbated lung inflammation that caused by allergen (79).

### Secondary Infection and Trained Immunity

**Figure 2:**
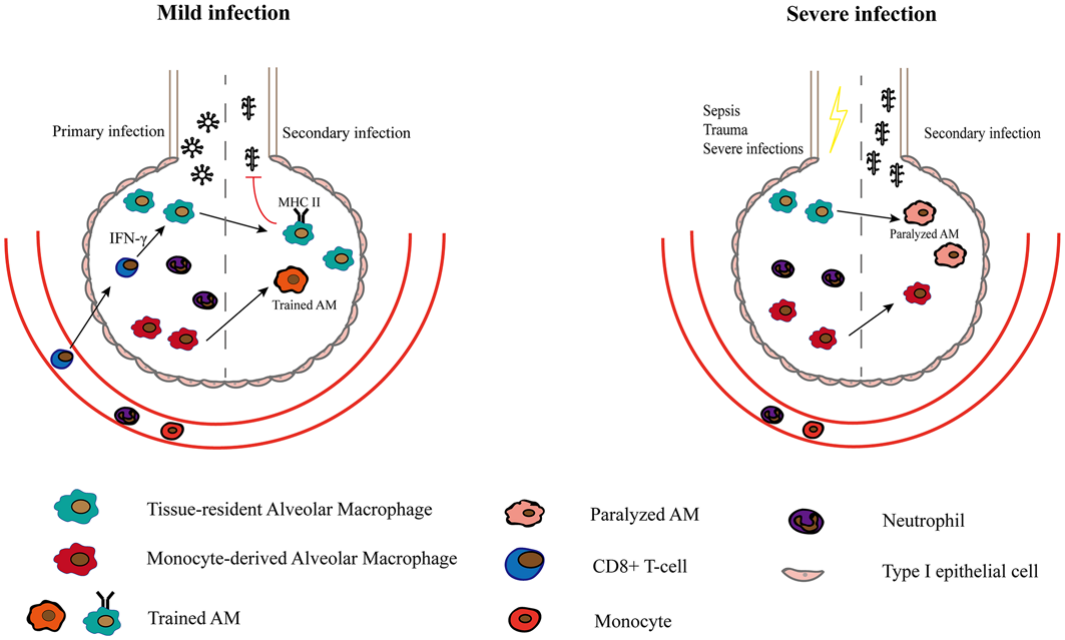
The role of Macrophages in secondary infection and trained immunity. After a mild infection caused by a specific pathogen such as adenoviruse, TR-AMs upregulate MHC II expression in response to CD8+ T lymphocytes and are rapidly activated in secondary infections (81). Mo-AMs that persists in the alveolar cavity after primary infection are also more likely to be activated and form part of trained AMs. After recovery from severe infections, TR-AMs are paralyzed with reduced phagocytosis and are more susceptible to secondary infections.

TR-AMs may also be related to secondary infection and trained immunity. After primary infection, the body may become more susceptible to bacterial infections. RSV infection stimulates the local production of growth arrest–specific 6 (Gas6), thereby converting TR-AMs into the M2 type, which impacts their ability to defend against secondary bacterial infections (80). After sepsis and severe trauma, TR-AMs are induced by secondary immunosuppressive signals of signal-regulatory protein α, and they are epigenetically altered, leading to long-term lung immunoparalysis (49). However, Yao et al. (81) found that respiratory viral infection induces long-lasting memory AMs that are programmed to express high MHC II levels, increase glycolytic metabolism and produce more neutrophil chemokines. These memory AMs are induced by CD8^+^ T cells (81). After influenza infection, some TR-AMs were replaced by Mo-AMs, which can protect the body from *Streptococcus pneumoniae* infection (42). This phenomenon can also be observed in a model where the pathogen of the first infection is bacteria. After recovery from *Streptococcus pneumoniae* infection, TR-AMs were reprogrammed and protected against another pneumococcal serotype (82). It is unclear why TR-AMs exhibit a completely different phenotype and function after the initial infection. The severity of the initial infection may be one of the reasons. Severe systematic infections such as sepsis can cause AMs to develop immune paralysis states where the phagocytosis and proinflammatory ability of AMs are impaired (83, 84). While some local mild infections can make AMs more likely to be activated in subsequent infections (81, 82) (**Figure 2**). However, both the protective effect and dysfunction of TR-AMs may be restricted to a particular model. Further investigations are required to determine how different pathogens induce unique microenvironments and how these microenvironments differentially affect AMs and lead to long-term changes in AM.

## Monocyte-Derived Alveolar Macrophages: Fanning the Flames

In homeostasis, two types of monocytes exist in the lung as follows: classical monocytes (Ly6C^hi^CX3CR1^lo–mid^CCR2^+^) and non-classical/patrolling monocytes (Ly6C^lo^CX3CR1^hi^CCR2^-^) (85). Non-classical monocytes primarily function to scavenge damaged cells and debris from the luminal side of the vascular endothelium as well as the parenchyma of tissues, while classical monocytes are the main recruited monocytes in inflammation (85, 86). During inflammation or injury, TR-AMs, epithelial cells and other innate cells release cytokines and chemokines, and classical monocytes are then recruited to the lungs where they differentiate into macrophages (**Figure 1**). Mo-AMs have a limited ability of self-maintenance (40, 87) and may undergo apoptosis at the late stage of inflammation (26). The remaining Mo-AMs can acquire the phenotype of AMs and exist for a long time, while some expressed genes still show changes. In addition to inflammation and injury states, Mo-AMs may also contribute to the AM pool in some steady states. Fate-mapping studies have shown that there is a substantial contribution of monocytes to the AM compartment in older mice (30, 88). This might be due to age-related depletion of TR-AMs (45, 47).

Local microenvironmental cues generated by tissue cells are increasingly recognized as critical determinants of resident macrophage identity. Newly recruited Mo-AMs are more plastic than TR-AMs and can be more easily instructed by the local microenvironment. Due to their monocyte origin, Mo-AMs are more likely to be remodelled by the microenvironment than TR-AMs. Newly arrived Mo-AMs may quickly acquire an inflammatory phenotype in an inflamed lung and further promote the development of inflammation. In the late stage of lung injury, a resolving environment can also instruct Mo-AMs to a pro-resolving phenotype and promote the resolution of inflammation. Similarly, the pro-fibrotic effect of Mo-AMs may be due to the early changes of the lung microenvironment, which imprint a pro-fibrotic phenotype on Mo-AMs.

### Infections and Lung Injury

In pulmonary viral and bacterial infections, Mo-AMs play an important role in the clearance of pathogens through phagocytosis and inflammatory responses. However, the strong proinflammatory effects of newly recruited Mo-AMs usually exacerbate lung injury. Mo-AMs can cause alveolar epithelial cell apoptosis and lung injury by releasing tumor necrosis factor-related apoptosis-inducing ligand and some inflammatory cytokines, such as TNF-α (89–92). The level of inflammatory Mo-AMs may also be related to the severity of diseases such as COVID-19 (93–95). The increased number of monocytes and Mo-AMs in COVID-19 patients may lead to cytokine storms, which can cause tissue damage, affect the adaptive immune response and increase mortality (96–99). In the LPS-induced lung injury model, different studies may have obtained opposite results on the role of monocytes and Mo-AMs, with some studies showing that they are important factors in causing injury and others finding that they promote recovery from inflammation, possibly because of the selection of time and choice of experimental method or mouse type (10, 11, 100).

At the later stage of infection, a gradual resolution in inflammation converts proinflammatory Mo-AMs to a pro-repair phenotype, which, in turn, promotes inflammatory resolution. Continuing persistence of inflammatory Mo-AMs may impair the process of recovery (92). However, it remains unknown how inflammatory Mo-AMs gradually disappear from the alveoli and whether apoptosis or migration occurs. The mechanism of how proinflammatory Mo-AMs transfer into a pro-repair phenotype is also under debate. Efferocytosis has been shown to promote macrophages to exhibit a proresolution phenotype. After phagocytosing too many apoptotic cells, the phenotype of macrophages further changes from the M2 type to a nonphagocytic CD11b-low phenotype, which plays a role in promoting inflammation resolution (58, 101). These macrophages have significantly different transcriptional characteristics than M2 macrophages and that they express high levels of IFNβ-related genes (102). This type of macrophage promotes the elimination of bacteria by secreting IFNβ, which promotes the apoptosis of inflammatory neutrophils through the STAT3 pathway, thereby enhancing efferocytosis and further promoting phenotypic changes in other macrophages (102). Because many studies on macrophage efferocytosis do not distinguish their origin, it remains unknown which type of macrophages play the main role of phagocytosis of apoptotic cells, whether their phagocytic ability is the same and which cells mainly promote the repair of damage. After recovery from infections, some Mo-AMs join the AM pool. These Mo-AMs display a unique functional, transcriptional and epigenetic profile, and they produce increased IL-6, which protects the lung from subsequent *Streptococcus pneumoniae* challenge (42). However, influenza-experienced resident AMs remain largely similar to naive AMs (42). Thus, the circumstances under which pathogens induce long-term changes in TR-AMs or substantial replenishment of different functional Mo-AMs remain to be explored.

### Type II Inflammation and Fibrosis

Studies have found that Mo-AMs can aggravate type II inflammation, which is contrary to TR-AMs. Depleting Mo-AMs can alleviate type II inflammation and fibrosis (25, 103). However, Machiels et al. (104) identified Mo-AMs as having a positive role in type II inflammation after long-term training of lung immunity. The Mo-AMs that replace TR-AMs can block the ability of dendritic cells to trigger an HDM-specific response by the TH2 subset of helper T cells (104). Importantly, the role of Mo-AMs in lung fibrosis has been gradually revealed. Using scRNA-seq, Aran et al. (105) found CX3CR1^+^ SiglecF^+^ macrophages to be a source of Pdgf-aa in the fibrotic niche. These CX3CR1^+^ SiglecF^+^ macrophages may be of monocyte origin and acquire a TR-AM profile (105). Fastrès et al. (106) also used scRNA-seq to characterize macrophage/monocyte cell populations in the BALF from dogs with canine idiopathic pulmonary fibrosis (CIPF) (107) and found that monocyte-derived macrophages were enriched in profibrotic genes in CIPF. Misharin et al. (6) found that during lung fibrosis, AMs are partially depleted and replaced by Mo-AMs. These Mo-AMs persist in the lung over the lifespan and drive lung fibrosis. The self-maintenance and persistence of these pathogenic Mo-AMs are controlled by macrophage colony-stimulating factor receptor signaling (7). Selective deletion of Mo-AMs can improve fibrosis (7, 8, 108). These studies indicate that monocyte-derived cells more easily acquire profibrotic phenotypes and, therefore, promote fibrosis. However, it remains unclear how profibrotic environments imprint Mo-AMs. Considering that many studies involve monocyte-derived macrophages containing monocyte-derived IMs, it remains unknown whether they act individually or together.

## Lung Interstitial Macrophages: Obscurity in the Past

### Physiological Characteristics

IMs, which are differentiated from AMs by their localization, remain less studied. Due to new transgenic tools and single-cell technology, the localization and function of IMs has become increasingly clear. Different subpopulations of IMs exist and reside in different anatomical sites. However, the exact locale of IMs is still not clear and requires additional studies. Gibbings et al. (109) identified the following three types of IMs by using flow cytometry in the steady state: CD11c^lo^ MHCII^lo^ IMs (IM1), CD11c^lo^ MHCII^hi^ IMs (IM2) and CD11c^+^ MHCII^hi^ IMs (IM3). All three IMs expressed high levels of CX3CR1 and and Csf1r (109). Compared to IM3 cells, IM1 and IM2 cells express higher levels of CD206, Lyve-1 and CD169 but lower levels of CCR2 and CD11c. BM chimeras and parabiotic mice demonstrate that IM3 more readily replenishes circulating precursor cells than IM1 and IM2 (109). Using CX3CR1-GFP reporter mice and immunostaining for MerTK, they found that IMs are located within the bronchial interstitium and not the alveolar interstitium (109). Chakarov et al. (110) identified two groups of IMs as follows: Lyve1^lo^ MHCII^hi^ and Lyve1^hi^ MHCII^lo^ IMs. Lyve1^lo^ MHCII^hi^ macrophages were mostly found surrounding the nerves and were mainly involved in inflammation and antigen presentation, while Lyve1^hi^ MHCII^lo^ macrophages were often closely associated with blood vessels and were mainly involved in wound healing and tissue repair. Schyns et al. (111) also found two subsets of IMs: CD206^+^ and CD206^−^IMs. CD206^+^ IMs mainly exist in bronchial interstitium and express high level of chemokines and anti-inflammation-related genes while CD206^−^ IMs are mainly exist in alveolar interstitium and express high level of antigen representation and proinflammation-related genes. Based on the above studies, at least two populations of IMs exist, namely, Lyve1^lo^ MHCII^hi^ CD206^−^ IMs and Lyve1^hi^ MHCII^lo^ CD206^+^ IMs, which reside in different areas of lung tissue and have distinct characteristics and functions (**Table 1**).

**Table 1:** Markers and functions of lung macrophages.

Cell type	Population	Markers	Functions	References
**TR-AM**		CD64^+^ MerTK^+^ F4/80^+^ SiglecF^hi^ CD11c^hi^ CD11b^lo^ CD206^hi^	homeostasis maintenance, pathogen phagocytosis, inflammation initiation, inflammation resolution	(5, 26, 42, 49)
**Mo-AM**		CD64^+^ MerTK^+^ F4/80^+^ SiglecF^-^ CD11c^-^ CD11b^hi^ CD206^lo^	pathogen phagocytosis, pro-inflammation, cytokines secretion	(8, 26, 42, 49)
**IM**	Lyve1^lo^ MHCII^hi^	CD64^+^ MerTK^+^ F4/80^+^ SiglecF^-^ CD11c^-^ CD11b^hi^ CD206^lo^ CX3CR1^hi^	inflammation and antigen presentation	(110, 111, 113)
	Lyve1^hi^ MHCII^lo^	CD64^+^ MerTK^+^ F4/80^+^ SiglecF^-^ CD11c^-^ CD11b^hi^ CD206^hi^ CX3CR1^lo^	wound healing and tissue repair	(110, 111, 113)

The origin of IMs is not clarified mainly due to the lack of reliable markers that could be used for lineage tracing experiments. Yolk sac macrophages and fetal liver monocytes may be the main origins of IMs before birth (112). Unlike TR-AMs, IMs can be gradually replaced by circulating monocytes after birth (112). Therefore, adult IMs may constitute a heterogeneous group of cells, comprising embryonically derived TR-IMs and bone marrow monocyte derived Mo-IMs. However, it remains unknown whether TR-IMs and Mo-IMs have different characteristics and functions in steady and pathogenic states similar to TR-AMs and Mo-AMs. Novel methods to distinguish these two types of IMs are needed. It also remains unclear whether there is a group of self-renewing IMs. Schyns et al. (111) found that CD206^+^ IMs have a longer life cycle, and CD206^−^ IMs may be derived from Ly6C^lo^ patrolling monocytes, which are defined as CD64^+^ CD16.2^+^ monocytes. Ural et al. (113) found a population of tissue-resident interstitial macrophages in the vicinity of sympathetic nerves in the bronchovascular bundle. These nerve- and airway-associated macrophages are derived from the yolk sac, are self-renewing and do not require CCR2+ monocytes for development or maintenance (113). Keerthivasan et al. (114) also identified a proliferative Ki67^+^ IM subpopulation using scRNA-seq. In contrast, using fate mapping and parabiotic mouse models, Chakarov et al. (110) demonstrated that both groups of IMs are replaced by blood monocytes after birth.

### Inflammation and Infections

IMs may take part in the inflammation process and regulate immune reactions. After intraperitoneal (i.p.) LPS administration, the proportion of IMs gradually increase during the course of inflammation (106). IMs and inflammatory monocytes (iMos) exhibit robust and largely overlapping changes in gene expression (106). In addition to proinflammatory cytokines, IMs are quickly induced to express genes for anti-inflammatory cytokines, active oxygen scavengers and matrix metallopeptidases after i.p. LPS administration (106). In contrast to i.p. delivery, AMs are the most responsive lung macrophages after intranasal (i.n.) LPS administration with few acute changes in gene expression observed in IMs and iMos (106). The lung tissue environment may also have an impact on the immune regulation function of IMs, and IMs may interact with other nearby cells. In response to inflammatory injury, IMs switch to an anti-inflammatory phenotype to maintain lung homeostasis, which is regulated by Rspondin3 secreted by endothelial cells (115). IMs also have functions in type II inflammation and parasite infections. IMs can expand under the stimulation of bacterial CpG DNA and produce IL-10 to reduce allergic reactions (116). IMs have also been found to produce high levels of IL-10 to inhibit LPS-induced maturation and migration of DCs (117). *Nippostrongylus brasiliensis* infection induces expansion of RELMα^+^ lung IMs but not AMs. RELMα^+^ lung interstitial macrophages are necessary for reducing severe lung injury in primary and secondary infection (118).

### Fibrosis

The role of IMs in lung fibrosis has been gradually discovered, but the exact role is still unclear. During lung fibrosis, both interstitial and alveolar macrophages are detected in clinical and preclinical radiation-induced lung fibrosis (RIF). Depletion of IMs using colony-stimulating factor receptor-1 (CSF1R) neutralizing antibody effectively reduces fibrosis *in vivo*, while depletion of TR-AMs has no effect on the RIF score (119). The arginase-1 expression level in IMs is significantly higher than that in AMs both in the physiological state and in RIF (119). Other studies on monocyte-derived macrophages indicate a possible role of IMs in promoting fibrosis. Depletion of SiglecF^lo^ CD11b^hi^ macrophages, depletion of Cx3cr1^+^ cells or use of CCR2-deficient mice can reduce pulmonary fibrosis (7, 120–122). All these cell depletion methods may cause the loss of IMs. However, Chakarov et al. (110) found that the absence of Lyve1^hi^MHCII^lo^ IMs exacerbates experimental lung and heart fibrosis, demonstrating their protective role in fibrosis. This result suggests the role of origin in IM functions. Lyve1^hi^MHCII^lo^ IMs that express high levels of CD206 and CD169 are more likely to be resident IMs and may play a positive role in fibrosis. However, monocyte-derived IMs may have a profibrotic role similar to Mo-AMs. It remains unknown whether the origin determines the destiny of IMs.

## Targeting Macrophages in Lung Diseases

Targeting macrophages has been studied in tumor and autoimmune diseases (123–125). Targeting tumor-associated macrophages and related factors has been partially applied in the clinic (126). In various diseases of the lung, targeting macrophages may be a new strategy, especially for infectious diseases such as COVID-19 and pulmonary fibrosis. Macrophage-targeted antibiotics or prodrugs to the lung are used to treat specific pathogens that infect macrophages (127, 128). Because Mo-AMs are important pathogenic factors in severe infections and pulmonary fibrosis, direct or indirect targeting of Mo-AMs may be a new approach for the future treatment of infectious and fibrotic diseases. Direct clearance of Mo-AMs in mice can effectively reduce infection-induced lung injury and pulmonary fibrosis, and this approach can be optimized for clinical application. Since direct clearance of monocytes or Mo-AMs may have an unexpected impact on the human body, indirect targeting of monocytes and Mo-AMs may be a better option. Researchers can block certain factors or pathways to reduce the proinflammatory nature of macrophages or to promote their conversion to an anti-inflammatory phenotype. For example, in the treatment of COVID-19, targeting GM-CSF can reduce the proinflammatory properties of macrophages (129). Moreover, blocking the CCL2-CCR2 axis may be another method to stop the extensive recruitment of monocytes and Mo-AMs (130). In addition, compared to that of Mo-AMs, the number of TR-AMs is significantly reduced in severe infection and fibrosis. Immunomodulatory effects may be achieved by supplementing TR-AMs or promoting their proliferation, and timely restoration of TR-AMs also prevents the development of fibrosis after Mo-AMs occupy the niche.

## Concluding Remarks

Recent findings have revealed different subtypes of lung macrophages that play important roles in both homeostatic and disease states. TR-AMs are long-lived cells shaped by the microenvironment and have immunosuppressive functions in the steady state and less plasticity in the defense state. TR-AMs play an indispensable role in fighting pathogens as they activate the inflammatory response in the early stages and promote the recovery of inflammation in the late stages. However, whether TR-AMs are truly self-renewing and whether TR-AMs have motility properties remain controversial. The differences between mouse AMs and human AMs are unknown, and the origin of human TR-AMs and the composition of the human AM pool remain to be further discovered. It is also unclear whether sustained pathogen and dust exposure leads to a predominantly monocytic origin of human lung macrophages. Derived from monocytes, Mo-AMs are more easily instructed by the environment than TR-AMs, and they are associated with cytokine storms and immune imbalance in severe infections (e.g., COVID-19). Timely regression of inflammatory macrophages and their conversion to an anti-inflammatory phenotype is essential for normal recovery from inflammation. Thus, interfering with excess inflammation, Mo-AMs may be a potential mechanism to correct the immune imbalance. In the recovery period, how Mo-AMs convert to a pro-resolving phenotype and whether they undergo apoptosis or migration are unknown. After primary infection, the function of Mo-AMs in trained immunity are other controversial issues. Considering the pro-fibrotic function of Mo-AMs, it remains to be determined whether Mo-AMs infiltrating the infected lung receive different instructions than the those infiltrating a fibrotic lung, and methods to change the microenvironment to alter Mo-AMs require more research in the future. Studies on IMs are lacking, and these cells may play essential roles in immune regulation, the type II inflammatory response and pulmonary fibrosis. It is important to accurately group IMs and determine their location and to clarify how the location and origin affect their function. It is also important to understand if IMs of different locations and origins have different functions in lung disease as well as how the microenvironment of lung tissue affects the function of IMs. When studying IMs, it is important to consider how to avoid contamination by other cells, such as Mo-AMs. In future studies, distinguishing different macrophages in lung infections and noninfectious diseases may help better understand macrophages and diseases.

## Author Contributions

All authors contributed to the study conception and design. Material preparation, data collection and analysis were performed by FH, KX, LX, and LT. The first draft of the manuscript was written by FH and XK and all authors commented on previous versions of the manuscript. All authors contributed to the article and approved the submitted version.

## Funding

This work was supported by Chinese PLA key project (BLB18J008).

## Conflict of Interest

The authors declare that the research was conducted in the absence of any commercial or financial relationships that could be construed as a potential conflict of interest.

## Publisher’s Note

All claims expressed in this article are solely those of the authors and do not necessarily represent those of their affiliated organizations, or those of the publisher, the editors and the reviewers. Any product that may be evaluated in this article, or claim that may be made by its manufacturer, is not guaranteed or endorsed by the publisher.

## References

[1] Byrne AJ Mathie SA Gregory LG Lloyd CM. Pulmonary Macrophages: Key Players in the Innate Defence of the Airways. Thorax (2015) 70(12):1189–96. doi: 10.1136/thoraxjnl-2015-207020 26286722

[2] Shi T Denney L An H Zheng Y. Alveolar and Lung Interstitial Macrophages: Definitions, Functions, and Roles in Lung Fibrosis. J Leukoc Biol (2021) 110(1):107–14. doi: 10.1002/JLB.3RU0720-418R 33155728

[3] Ogger PP Albers GJ Hewitt RJ O'Sullivan BJ Powell JE Calamita E. Itaconate Controls the Severity of Pulmonary Fibrosis. Sci Immunol (2020) 5(52):eabc1884. doi: 10.1126/sciimmunol.abc1884 33097591 PMC7116646

[4] Hussell T Bell TJ. Alveolar Macrophages: Plasticity in a Tissue-Specific Context. Nat Rev Immunol (2014) 14(2):81–93. doi: 10.1038/nri3600 24445666

[5] Guilliams M Svedberg FR. Does Tissue Imprinting Restrict Macrophage Plasticity? Nat Immunol (2021) 22(2):118–27. doi: 10.1038/s41590-020-00849-2 33462453

[6] Kulikauskaite J Wack A. Teaching Old Dogs New Tricks? The Plasticity of Lung Alveolar Macrophage Subsets. Trends Immunol (2020) 41(10):864–77. doi: 10.1016/j.it.2020.08.008 PMC7472979 32896485

[7] Joshi N Watanabe S Verma R Jablonski RP Chen C-I Cheresh P. A Spatially Restricted Fibrotic Niche in Pulmonary Fibrosis Is Sustained by M-CSF/M-CSFR Signalling in Monocyte-Derived Alveolar Macrophages. Eur Respir J (2020) 55(1):1900646. doi: 10.1183/13993003.00646-2019 31601718 PMC6962769

[8] Misharin AV Morales-Nebreda L Reyfman PA Cuda CM Walter JM McQuattie-Pimentel AC. Monocyte-Derived Alveolar Macrophages Drive Lung Fibrosis and Persist in the Lung Over the Life Span. J Exp Med (2017) 214(8):2387–404. doi: 10.1084/jem.20162152 PMC5551573 28694385

[9] Mould KJ Barthel L Mohning MP Thomas SM McCubbrey AL Danhorn T. Cell Origin Dictates Programming of Resident Versus Recruited Macrophages During Acute Lung Injury. Am J Respir Cell Mol Biol (2017) 57(3):294–306. doi: 10.1165/rcmb.2017-0061OC 28421818 PMC5625228

[10] Joshi JC Joshi B Rochford I Rayees S Akhter MZ Baweja S. SPHK2-Generated S1P in CD11b Macrophages Blocks STING to Suppress the Inflammatory Function of Alveolar Macrophages. Cell Rep (2020) 30(12):4096–109.e5. doi: 10.1016/j.celrep.2020.02.112 32209471 PMC7170050

[11] Herold S Tabar TS Janssen H Hoegner K Cabanski M Lewe-Schlosser P. Exudate Macrophages Attenuate Lung Injury by the Release of IL-1 Receptor Antagonist in Gram-Negative Pneumonia. Am J Respir Crit Care Med (2011) 183(10):1380–90. doi: 10.1164/rccm.201009-1431OC 21278303

[12] Liu X Boyer MA Holmgren AM Shin S. Legionella-Infected Macrophages Engage the Alveolar Epithelium to Metabolically Reprogram Myeloid Cells and Promote Antibacterial Inflammation. Cell Host Microbe (2020) 28(5):683–98.e6. doi: 10.1016/j.chom.2020.07.019 32841604 PMC9339267

[13] Westphalen K Gusarova GA Islam MN Subramanian M Cohen TS Prince AS. Sessile Alveolar Macrophages Communicate With Alveolar Epithelium to Modulate Immunity. Nature (2014) 506(7489):503–6. doi: 10.1038/nature12902 PMC4117212 24463523

[14] Lavine KJ Epelman S Uchida K Weber KJ Nichols CG Schilling JD. Distinct Macrophage Lineages Contribute to Disparate Patterns of Cardiac Recovery and Remodeling in the Neonatal and Adult Heart. Proc Natl Acad Sci USA (2014) 111(45):16029–34. doi: 10.1073/pnas.1406508111 PMC4234568 25349429

[15] Duffield JS Forbes SJ Constandinou CM Clay S Partolina M Vuthoori S. Selective Depletion of Macrophages Reveals Distinct, Opposing Roles During Liver Injury and Repair. J Clin Invest (2005) 115(1):56–65. doi: 10.1172/JCI200522675 15630444 PMC539199

[16] Zhang MZ Yao B Yang S Jiang L Wang S Fan X. CSF-1 Signaling Mediates Recovery From Acute Kidney Injury. J Clin Invest (2012) 122(12):4519–32. doi: 10.1172/JCI60363 PMC3533529 23143303

[17] Miron VE Boyd A Zhao JW Yuen TJ Ruckh JM Shadrach JL. M2 Microglia and Macrophages Drive Oligodendrocyte Differentiation During CNS Remyelination. Nat Neurosci (2013) 16(9):1211–8. doi: 10.1038/nn.3469 PMC3977045 23872599

[18] Shechter R Miller O Yovel G Rosenzweig N London A Ruckh J. Recruitment of Beneficial M2 Macrophages to Injured Spinal Cord Is Orchestrated by Remote Brain Choroid Plexus. Immunity (2013) 38(3):555–69. doi: 10.1016/j.immuni.2013.02.012 PMC4115271 23477737

[19] Zhou X Moore BB. Location or Origin? What Is Critical for Macrophage Propagation of Lung Fibrosis? Eur Respir J (2018) 51(3):1800103. doi: 10.1183/13993003.00103-2018 29496789 PMC6383715

[20] Hume PS Gibbings SL Jakubzick CV Tuder RM Curran-Everett D Henson PM. Localization of Macrophages in the Human Lung *via* Design-Based Stereology. Am J Respir Crit Care Med (2020) 201(10):1209–17. doi: 10.1164/rccm.201911-2105OC PMC7233346 32197050

[21] Desch AN Gibbings SL Goyal R Kolde R Bednarek J Bruno T. Flow Cytometric Analysis of Mononuclear Phagocytes in Nondiseased Human Lung and Lung-Draining Lymph Nodes. Am J Respir Crit Care Med (2016) 193(6):614–26. doi: 10.1164/rccm.201507-1376OC PMC4824940 26551758

[22] Mould KJ Jackson ND Henson PM Seibold M Janssen WJ. Single Cell RNA Sequencing Identifies Unique Inflammatory Airspace Macrophage Subsets. JCI Insight (2019) 4(5):e126556. doi: 10.1172/jci.insight.126556 PMC6483508 30721157

[23] Woods PS Kimmig LM Meliton AY Sun KA Tian Y O'Leary EM. Tissue Resident Alveolar Macrophages Do Not Rely on Glycolysis for LPS-Induced Inflammation. Am J Respir Cell Mol Biol (2019) 62(2):243–55. doi: 10.1165/rcmb.2019-0244OC PMC6993551 31469581

[24] Gibbings SL Goyal R Desch AN Leach SM Prabagar M Atif SM. Transcriptome Analysis Highlights the Conserved Difference Between Embryonic and Postnatal-Derived Alveolar Macrophages. Blood (2015) 126(11):1357–66. doi: 10.1182/blood-2015-01-624809 PMC4566811 26232173

[25] Zasłona Z Przybranowski S Wilke C van Rooijen N Teitz-Tennenbaum S Osterholzer JJ. Resident Alveolar Macrophages Suppress, Whereas Recruited Monocytes Promote, Allergic Lung Inflammation in Murine Models of Asthma. J Immunol (2014) 193(8):4245–53. doi: 10.4049/jimmunol.1400580 PMC4185233 25225663

[26] Janssen WJ Barthel L Muldrow A Oberley-Deegan RE Kearns MT Jakubzick C. Fas Determines Differential Fates of Resident and Recruited Macrophages During Resolution of Acute Lung Injury. Am J Respir Crit Care Med (2011) 184(5):547–60. doi: 10.1164/rccm.201011-1891OC PMC3175550 21471090

[27] Trapnell BC Whitsett JA Nakata K. Pulmonary Alveolar Proteinosis. N Engl J Med (2003) 349(26):2527–39. doi: 10.1056/NEJMra023226 14695413

[28] Nakamura A Ebina-Shibuya R Itoh-Nakadai A Muto A Shima H Saigusa D. Transcription Repressor Bach2 Is Required for Pulmonary Surfactant Homeostasis and Alveolar Macrophage Function. J Exp Med (2013) 210(11):2191–204. doi: 10.1084/jem.20130028 PMC3804940 24127487

[29] Roberts AW Lee BL Deguine J John S Shlomchik MJ Barton GM. Tissue-Resident Macrophages Are Locally Programmed for Silent Clearance of Apoptotic Cells. Immunity (2017) 47(5):913–27.e6. doi: 10.1016/j.immuni.2017.10.006 29150239 PMC5728676

[30] Gomez Perdiguero E Klapproth K Schulz C Busch K Azzoni E Crozet L. Tissue-Resident Macrophages Originate From Yolk-Sac-Derived Erythro-Myeloid Progenitors. Nature (2015) 518(7540):547–51. doi: 10.1038/nature13989 PMC5997177 25470051

[31] Hoeffel G Chen J Lavin Y Low D Almeida FF See P. C-Myb(+) Erythro-Myeloid Progenitor-Derived Fetal Monocytes Give Rise to Adult Tissue-Resident Macrophages. Immunity (2015) 42(4):665–78. doi: 10.1016/j.immuni.2015.03.011 PMC4545768 25902481

[32] Guilliams M De Kleer I Henri S Post S Vanhoutte L De Prijck S. Alveolar Macrophages Develop From Fetal Monocytes That Differentiate Into Long-Lived Cells in the First Week of Life *via* GM-CSF. J Exp Med (2013) 210(10):1977–92. doi: 10.1084/jem.20131199 PMC3782041 24043763

[33] Yu X Buttgereit A Lelios I Utz SG Cansever D Becher B. The Cytokine Tgf-β Promotes the Development and Homeostasis of Alveolar Macrophages. Immun (2017) 47(5):903–12.e4. doi: 10.1016/j.immuni.2017.10.007 29126797

[34] Schneider C Nobs SP Kurrer M Rehrauer H Thiele C Kopf M. Induction of the Nuclear Receptor PPAR-γ by the Cytokine GM-CSF Is Critical for the Differentiation of Fetal Monocytes Into Alveolar Macrophages. Nat Immunol (2014) 15(11):1026–37. doi: 10.1038/ni.3005 25263125

[35] Shibata Y Berclaz PY Chroneos ZC Yoshida M Whitsett JA Trapnell BC. GM-CSF Regulates Alveolar Macrophage Differentiation and Innate Immunity in the Lung Through PU.1. Immunity (2001) 15(4):557–67. doi: 10.1016/S1074-7613(01)00218-7 11672538

[36] Naessens T Schepens B Smet M Pollard C Van Hoecke L De Beuckelaer A. GM-CSF Treatment Prevents Respiratory Syncytial Virus-Induced Pulmonary Exacerbation Responses in Postallergic Mice by Stimulating Alveolar Macrophage Maturation. J Allergy Clin Immunol (2016) 137(3):700–9.e9. doi: 10.1016/j.jaci.2015.09.031 26560044

[37] Rauschmeier R Gustafsson C Reinhardt A A-Gonzalez N Tortola L Cansever D. Bhlhe40 and Bhlhe41 Transcription Factors Regulate Alveolar Macrophage Self-Renewal and Identity. EMBO J (2019) 38(19):e101233. doi: 10.15252/embj.2018101233 31414712 PMC6769426

[38] Yao Y Liu Q Adrianto I Wu X Glassbrook J Khalasawi N. Histone Deacetylase 3 Controls Lung Alveolar Macrophage Development and Homeostasis. Nat Commun (2020) 11(1):3822. doi: 10.1038/s41467-020-17630-6 32732898 PMC7393351

[39] Cohen M Giladi A Gorki AD Solodkin DG Zada M Hladik A. Lung Single-Cell Signaling Interaction Map Reveals Basophil Role in Macrophage Imprinting. Cell (2018) 175(4):1031–44.e18. doi: 10.1016/j.cell.2018.09.009 30318149

[40] Hashimoto D Chow A Noizat C Teo P Beasley MB Leboeuf M. Tissue-Resident Macrophages Self-Maintain Locally Throughout Adult Life With Minimal Contribution From Circulating Monocytes. Immunity (2013) 38(4):792–804. doi: 10.1016/j.immuni.2013.04.004 23601688 PMC3853406

[41] Soucie EL Weng Z Geirsdóttir L Molawi K Maurizio J Fenouil R. Lineage-Specific Enhancers Activate Self-Renewal Genes in Macrophages and Embryonic Stem Cells. Science (2016) 351(6274):aad5510. doi: 10.1126/science.aad5510 26797145 PMC4811353

[42] Aegerter H Kulikauskaite J Crotta S Patel H Kelly G Hessel EM. Influenza-Induced Monocyte-Derived Alveolar Macrophages Confer Prolonged Antibacterial Protection. Nat Immunol (2020) 21(2):145–57. doi: 10.1038/s41590-019-0568-x PMC6983324 31932810

[43] Lai SM Sheng J Gupta P Renia L Duan K Zolezzi F. Organ-Specific Fate, Recruitment, and Refilling Dynamics of Tissue-Resident Macrophages During Blood-Stage Malaria. Cell Rep (2018) 25(11):3099–109.e3. doi: 10.1016/j.celrep.2018.11.059 30540942

[44] Evren E Ringqvist E Tripathi KP Sleiers N Rives IC Alisjahbana A. Distinct Developmental Pathways From Blood Monocytes Generate Human Lung Macrophage Diversity. Immunity (2020) 54(2):259–75.e7. doi: 10.1016/j.immuni.2020.12.003 33382972

[45] Byrne AJ Powell JE O’sullivan BJ Ogger PP Hoffland A Cook J. Dynamics of Human Monocytes and Airway Macrophages During Healthy Aging and After Transplant. J Exp Med (2020) 217(3):e20191236. doi: 10.1084/jem.20191236 31917836 PMC7062517

[46] Mould KJ Moore CM Mcmanus SA McCubbrey AL McClendon JD Griesmer CL. Airspace Macrophages and Monocytes Exist in Transcriptionally Distinct Subsets in Healthy Adults. Am J Respir Crit Care Med (2020) 203(8):946–56. doi: 10.1164/rccm.202005-1989OC PMC8048748 33079572

[47] Wong CK Smith CA Sakamoto K Kaminski N Koff JL Goldstein DR. Aging Impairs Alveolar Macrophage Phagocytosis and Increases Influenza-Induced Mortality in Mice. J Immunol (Baltimore Md.: 1950) (2017) 199(3):1060–8. doi: 10.4049/jimmunol.1700397 PMC5557035 28646038

[48] O’beirne SL Kikkers SA Oromendia C Salit J Rostmai MR Ballman KV. Alveolar Macrophage Immunometabolism and Lung Function Impairment in Smoking and Chronic Obstructive Pulmonary Disease. Am J Respir Crit Care Med (2020) 201(6):735–9. doi: 10.1164/rccm.201908-1683LE PMC7068819 31751151

[49] Roquilly A Jacqueline C Davieau M Mollé A Sadek A Fourgeux C. Alveolar Macrophages Are Epigenetically Altered After Inflammation, Leading to Long-Term Lung Immunoparalysis. Nat Immunol (2020) 21(6):636–48. doi: 10.1038/s41590-020-0673-x 32424365

[50] Neupane AS Willson M Chojnacki AK Vargas ESCF Morehouse C Carestia A. Patrolling Alveolar Macrophages Conceal Bacteria From the Immune System to Maintain Homeostasis. Cell (2020) 183(1):110–25.e11. doi: 10.1016/j.cell.2020.08.020 32888431

[51] Beck-Schimmer B Schwendener R Pasch T Reyes L Booy C Schimmer RC. Alveolar Macrophages Regulate Neutrophil Recruitment in Endotoxin-Induced Lung Injury. Respir Res (2005) 6(1):61. doi: 10.1186/1465-9921-6-61 15972102 PMC1188075

[52] Pribul PK Harker J Wang B Wang H Tregoning JS Schwarze J. Alveolar Macrophages Are a Major Determinant of Early Responses to Viral Lung Infection But Do Not Influence Subsequent Disease Development. J Virol (2008) 82(9):4441–8. doi: 10.1128/JVI.02541-07 PMC2293049 18287232

[53] Xu-Vanpala S Deerhake ME Wheaton JD Parker ME Juvvadi PR MacIver N. Functional Heterogeneity of Alveolar Macrophage Population Based on Expression of CXCL2. Sci Immunol (2020) 5(50):eaba7350. doi: 10.1126/sciimmunol.aba7350 32769172 PMC7717592

[54] Herold S Mayer K Lohmeyer J. Acute Lung Injury: How Macrophages Orchestrate Resolution of Inflammation and Tissue Repair. Front Immunol (2011) 2:65. doi: 10.3389/fimmu.2011.00065 22566854 PMC3342347

[55] Wynn TA Vannella KM. Macrophages in Tissue Repair, Regeneration, and Fibrosis. Immunity (2016) 44(3):450–62. doi: 10.1016/j.immuni.2016.02.015 PMC4794754 26982353

[56] Uderhardt S Herrmann M Oskolkova OV Aschermann S Bicker W Ipseiz N. 12/15-Lipoxygenase Orchestrates the Clearance of Apoptotic Cells and Maintains Immunologic Tolerance. Immunity (2012) 36(5):834–46. doi: 10.1016/j.immuni.2012.03.010 22503541

[57] Mukundan L Odegaard JI Morel CR Heredia JE Mwangi JW Ricardo-Gonzalez RR. PPAR-Delta Senses and Orchestrates Clearance of Apoptotic Cells to Promote Tolerance. Nat Med (2009) 15(11):1266–72. doi: 10.1038/nm.2048 PMC2783696 19838202

[58] Ariel A Serhan CN. New Lives Given by Cell Death: Macrophage Differentiation Following Their Encounter With Apoptotic Leukocytes During the Resolution of Inflammation. Front Immunol (2012) 3:4. doi: 10.3389/fimmu.2012.00004 22566890 PMC3342362

[59] Branchett WJ Cook J Oliver RA Bruno N Walker SA Stölting H. Airway Macrophage-Intrinsic TGF-β1 Regulates Pulmonary Immunity During Early Life Allergen Exposure. J Allergy Clin Immunol (2021) 147(5):1892–906. doi: 10.1016/j.jaci.2021.01.026 PMC8098862 33571538

[60] Cakarova L Marsh LM Wilhelm J Mayer K Grimminger F Seeger W. Macrophage Tumor Necrosis Factor-Alpha Induces Epithelial Expression of Granulocyte-Macrophage Colony-Stimulating Factor: Impact on Alveolar Epithelial Repair. Am J Respir Crit Care Med (2009) 180(6):521–32. doi: 10.1164/rccm.200812-1837OC 19590023

[61] Huynh M-LN Fadok VA Henson PM. Phosphatidylserine-Dependent Ingestion of Apoptotic Cells Promotes TGF-Beta1 Secretion and the Resolution of Inflammation. J Clin Invest (2002) 109(1):41–50. doi: 10.1172/JCI0211638 11781349 PMC150814

[62] Grégoire M Uhel F Lesouhaitier M Gacouin A Guirriec M Mourcin F. Impaired Efferocytosis and Neutrophil Extracellular Trap Clearance by Macrophages in ARDS. . Eur Respir J (2018) 52(2):1702590. doi: 10.1183/13993003.02590-2017 29946009

[63] Jondle CN Gupta K Mishra BB Sharma J. Klebsiella Pneumoniae Infection of Murine Neutrophils Impairs Their Efferocytic Clearance by Modulating Cell Death Machinery. PloS Pathog (2018) 14(10):e1007338. doi: 10.1371/journal.ppat.1007338 30273394 PMC6181436

[64] Cohen TS Jones-Nelson O Hotz M Cheng L Miller LS Suzich J. S. Aureus Blocks Efferocytosis of Neutrophils by Macrophages Through the Activity of Its Virulence Factor Alpha Toxin. Sci Rep (2016) 6:35466. doi: 10.1038/srep35466 27739519 PMC5064327

[65] Martin FJ Parker D Harfenist BS Soong G Prince A. Participation of CD11c(+) Leukocytes in Methicillin-Resistant Staphylococcus Aureus Clearance From the Lung. Infect Immun (2011) 79(5):1898–904. doi: 10.1128/IAI.01299-10 PMC3088152 21402768

[66] Broug-Holub E Toews GB Van Iwaarden JF Strieter RM Kunkel SL Paine R. Alveolar Macrophages Are Required for Protective Pulmonary Defenses in Murine Klebsiella Pneumonia: Elimination of Alveolar Macrophages Increases Neutrophil Recruitment But Decreases Bacterial Clearance and Survival. Infect Immun (1997) 65(4):1139–46. doi: 10.1128/iai.65.4.1139-1146.1997 PMC175109 9119443

[67] Narasaraju T Yang E Samy RP Ng HH Poh WP Liew AA. Excessive Neutrophils and Neutrophil Extracellular Traps Contribute to Acute Lung Injury of Influenza Pneumonitis. Am J Pathol (2011) 179(1):199–210. doi: 10.1016/j.ajpath.2011.03.013 21703402 PMC3123873

[68] Cohen TS Prince AS. Activation of Inflammasome Signaling Mediates Pathology of Acute P. Aeruginosa Pneumonia. J Clin Invest (2013) 123(4):1630–7. doi: 10.1172/JCI66142 PMC3613922 23478406

[69] Santos LD Antunes KH Muraro SP de Souza GF da Silva AG de Souza Felipe J. TNF-Mediated Alveolar Macrophage Necroptosis Drives Disease Pathogenesis During Respiratory Syncytial Virus Infection. Eur Respir J (2020) 57(6):2003764. doi: 10.1183/13993003.03764-2020 PMC8209485 33303545

[70] Cohen SB Gern BH Delahaye JL Adams KN Plumlee CR Winkler JK. Alveolar Macrophages Provide an Early Mycobacterium Tuberculosis Niche and Initiate Dissemination. Cell Host Microbe (2018) 24(3):439–46.e4. doi: 10.1016/j.chom.2018.08.001 30146391 PMC6152889

[71] Eyal FG Hamm CR Parker JC. Reduction in Alveolar Macrophages Attenuates Acute Ventilator Induced Lung Injury in Rats. Intensive Care Med (2007) 33(7):1212–8. doi: 10.1007/s00134-007-0651-x 17468847

[72] Fahy JV. Type 2 Inflammation in Asthma–Present in Most, Absent in Many. Nat Rev Immunol (2015) 15(1):57–65. doi: 10.1038/nri3786 25534623 PMC4390063

[73] Lou H Huang Y Chen H Wang Y Zhang L Wang C. M2 Macrophages Correlated With Symptom Severity and Promote Type 2 Inflammation in Allergic Rhinitis. Allergy (2019) 74(11):2255–7. doi: 10.1111/all.13852 31059133

[74] Van Dyken SJ Locksley RM. Interleukin-4- and Interleukin-13-Mediated Alternatively Activated Macrophages: Roles in Homeostasis and Disease. Annu Rev Immunol (2013) 31:317–43. doi: 10.1146/annurev-immunol-032712-095906 PMC3606684 23298208

[75] Svedberg FR Brown SL Krauss MZ Campbell L Sharpe C Clausen M. The Lung Environment Controls Alveolar Macrophage Metabolism and Responsiveness in Type 2 Inflammation. Nat Immunol (2019) 20(5):571–80. doi: 10.1038/s41590-019-0352-y PMC8381729 30936493

[76] Miki H Pei H Gracias DT Linden J Croft M. Clearance of Apoptotic Cells by Lung Alveolar Macrophages Prevents Development of House Dust Mite-Induced Asthmatic Lung Inflammation. J Allergy Clin Immunol (2020) 147(3):1087–92.e3. doi: 10.1016/j.jaci.2020.10.005 33065121 PMC7940554

[77] Mathie SA Dixon KL Walker SA Tyrrell V Mondhe M O'Donnell VB. Alveolar Macrophages Are Sentinels of Murine Pulmonary Homeostasis Following Inhaled Antigen Challenge. Allergy (2015) 70(1):80–9. doi: 10.1111/all.12536 PMC4283732 25331546

[78] Soroosh P Doherty TA Duan W Mehta AK Choi H Adams YF. Lung-Resident Tissue Macrophages Generate Foxp3+ Regulatory T Cells and Promote Airway Tolerance. J Exp Med (2013) 210(4):775–88. doi: 10.1084/jem.20121849 PMC3620360 23547101

[79] Zhou Y Do DC Ishmael FT Squadrito ML Tang HM Tang HL. Mannose Receptor Modulates Macrophage Polarization and Allergic Inflammation Through MiR-511-3p. J Allergy Clin Immunol (2018) 141(1):350–64.e8. doi: 10.1016/j.jaci.2017.04.049 28629744 PMC5944850

[80] Shibata T Makino A Ogata R Nakamura S Ito T Nagata K. Respiratory Syncytial Virus Infection Exacerbates Pneumococcal Pneumonia *via* Gas6/Axl-Mediated Macrophage Polarization. J Clin Invest (2020) 130(6):3021–37. doi: 10.1172/JCI125505 PMC7260035 32364537

[81] Yao Y Jeyanathan M Haddadi S Barra NG Vaseghi-Shanjani M Damjanovic D. Induction of Autonomous Memory Alveolar Macrophages Requires T Cell Help and Is Critical to Trained Immunity. Cell (2018) 175(6):1634–50.e17. doi: 10.1016/j.cell.2018.09.042 30433869

[82] Guillon A Arafa EI Barker KA Belkina AC Martin IM Shenoy AT. Pneumonia Recovery Reprograms the Alveolar Macrophage Pool. JCI Insight (2020) 5(4):e133042. doi: 10.1172/jci.insight.133042 PMC7101156 31990682

[83] Hiruma T Tsuyuzaki H Uchida K Trapnell BC Yamamura Y Kusakabe Y. IFN-β Improves Sepsis-Related Alveolar Macrophage Dysfunction and Postseptic Acute Respiratory Distress Syndrome-Related Mortality. Am J Respir Cell Mol Biol (2018) 59(1):45–55. doi: 10.1165/rcmb.2017-0261OC 29365277 PMC6835072

[84] Pahuja M Tran C Wang H Yin K. Alveolar Macrophage Suppression in Sepsis Is Associated With High Mobility Group Box 1 Transmigration. Shock (2008) 29(6):754–60. doi: 10.1097/SHK.0b013e31815d0c8f 18004227

[85] Epelman S Lavine KJ Randolph GJ. Origin and Functions of Tissue Macrophages. Immunity (2014) 41(1):21–35. doi: 10.1016/j.immuni.2014.06.013 25035951 PMC4470379

[86] Auffray C Fogg D Garfa M Elain G Join-Lambert O Kayal S. Monitoring of Blood Vessels and Tissues by a Population of Monocytes With Patrolling Behavior. Science (2007) 317(5838):666–70. doi: 10.1126/science.1142883 17673663

[87] Scott CL Henri S Guilliams M. Mononuclear Phagocytes of the Intestine, the Skin, and the Lung. Immunol Rev (2014) 262(1):9–24. doi: 10.1111/imr.12220 25319324

[88] Liu Z Gu Y Chakarov S Bleriot C Kwok I Chen X. Fate Mapping *via* Ms4a3-Expression History Traces Monocyte-Derived Cells. Cell (2019) 178(6):1509–25.e19. doi: 10.1016/j.cell.2019.08.009 31491389

[89] Herold S Steinmueller M Von Wulffen W Cakarova L Pinto R Pleschka S. Lung Epithelial Apoptosis in Influenza Virus Pneumonia: The Role of Macrophage-Expressed TNF-Related Apoptosis-Inducing Ligand. J Exp Med (2008) 205(13):3065–77. doi: 10.1084/jem.20080201 PMC2605231 19064696

[90] Lin KL Suzuki Y Nakano H Ramsburg E Gunn MD. CCR2+ Monocyte-Derived Dendritic Cells and Exudate Macrophages Produce Influenza-Induced Pulmonary Immune Pathology and Mortality. J Immunol (Baltimore Md.: 1950) (2008) 180(4):2562–72. doi: 10.4049/jimmunol.180.4.2562 18250467

[91] Teijaro JR Walsh KB Rice S Rosen H Oldstone MB. Mapping the Innate Signaling Cascade Essential for Cytokine Storm During Influenza Virus Infection. Proc Natl Acad Sci USA (2014) 111(10):3799–804. doi: 10.1073/pnas.1400593111 PMC3956176 24572573

[92] Pernet E Downey J Vinh DC Powell WS Divangahi M. Leukotriene B(4)-Type I Interferon Axis Regulates Macrophage-Mediated Disease Tolerance to Influenza Infection. Nat Microbiol (2019) 4(8):1389–400. doi: 10.1038/s41564-019-0444-3 31110361

[93] Liao M Liu Y Yuan J Wen Y Xu G Zhao J. Single-Cell Landscape of Bronchoalveolar Immune Cells in Patients With COVID-19. Nat Med (2020) 26(6):842–4. doi: 10.1038/s41591-020-0901-9 32398875

[94] Wauters E Van Mol P Garg AD Jansen S Van Herck Y Vanderbeke L. Discriminating Mild From Critical COVID-19 by Innate and Adaptive Immune Single-Cell Profiling of Bronchoalveolar Lavages. Cell Res (2021) 31(3):272–90. doi: 10.1101/2020.07.09.196519 PMC8027624 33473155

[95] Ren X Wen W Fan X Hou W Su B Cai P. COVID-19 Immune Features Revealed by a Large-Scale Single-Cell Transcriptome Atlas. Cell (2021) 184(7):1895–913.e19. doi: 10.1016/j.cell.2021.01.053 33657410 PMC7857060

[96] Bost P Giladi A Liu Y Bendjelal Y Xu G David E. Host-Viral Infection Maps Reveal Signatures of Severe COVID-19 Patients. Cell (2020) 181(7):1475–88.e12. doi: 10.1016/j.cell.2020.05.006 32479746 PMC7205692

[97] Grant RA Morales-Nebreda L Markov NS Swaminathan S Querrey M Guzman ER. Circuits Between Infected Macrophages and T Cells in SARS-CoV-2 Pneumonia. Nature (2021) 590(7847):635–41. doi: 10.1038/s41586-020-03148-w PMC7987233 33429418

[98] Szabo PA Dogra P Gray JI Wells SB Connors TJ Weisberg SP. Longitudinal Profiling of Respiratory and Systemic Immune Responses Reveals Myeloid Cell-Driven Lung Inflammation in Severe COVID-19. Immunity (2021) 54(4):797–814.e6. doi: 10.1016/j.immuni.2021.03.005 33765436 PMC7951561

[99] Schultze JL Aschenbrenner AC. COVID-19 and the Human Innate Immune System. Cell (2021) 184(7):1671–92. doi: 10.1016/j.cell.2021.02.029 PMC7885626 33743212

[100] Dhaliwal K Scholefield E Ferenbach D Gibbons M Duffin R Dorward DA. Monocytes Control Second-Phase Neutrophil Emigration in Established Lipopolysaccharide-Induced Murine Lung Injury. Am J Respir Crit Care Med (2012) 186(6):514–24. doi: 10.1164/rccm.201112-2132OC PMC3480527 22822022

[101] Schif-Zuck S Gross N Assi S Rostoker R Serhan CN Ariel A. Saturated-Efferocytosis Generates Pro-Resolving CD11b Low Macrophages: Modulation by Resolvins and Glucocorticoids. Eur J Immunol (2011) 41(2):366–79. doi: 10.1002/eji.201040801 PMC3082320 21268007

[102] Kumaran Satyanarayanan S El Kebir D Soboh S Butenko S Sekheri M Saadi J. IFN-β Is a Macrophage-Derived Effector Cytokine Facilitating the Resolution of Bacterial Inflammation. Nat Commun (2019) 10(1):3471. doi: 10.1038/s41467-019-10903-9 31375662 PMC6677895

[103] Borthwick LA Barron L Hart KM Vannella KM Thompson RW Oland S. Macrophages Are Critical to the Maintenance of IL-13-Dependent Lung Inflammation and Fibrosis. Mucosal Immunol (2016) 9(1):38–55. doi: 10.1038/mi.2015.34 25921340 PMC4626445

[104] Machiels B Dourcy M Xiao X Javaux J Mesnil C Sabatel C. A Gammaherpesvirus Provides Protection Against Allergic Asthma by Inducing the Replacement of Resident Alveolar Macrophages With Regulatory Monocytes. Nat Immunol (2017) 18(12):1310–20. doi: 10.1038/ni.3857 29035391

[105] Aran D Looney AP Liu L Wu E Fong V Hsu A. Reference-Based Analysis of Lung Single-Cell Sequencing Reveals a Transitional Profibrotic Macrophage. Nat Immunol (2019) 20(2):163–72. doi: 10.1038/s41590-018-0276-y PMC6340744 30643263

[106] Sajti E Link VM Ouyang Z Spann NJ Westin E Romanoski CE. Transcriptomic and Epigenetic Mechanisms Underlying Myeloid Diversity in the Lung. Nat Immunol (2020) 21(2):221–31. doi: 10.1038/s41590-019-0582-z PMC7667722 31959980

[107] Fastrès A Pirottin D Fievez L Tutunaru AC Bolen G Merveille AC. Identification of Pro-Fibrotic Macrophage Populations by Single-Cell Transcriptomic Analysis in West Highland White Terriers Affected With Canine Idiopathic Pulmonary Fibrosis. Front Immunol (2020) 11:611749. doi: 10.3389/fimmu.2020.611749 33384697 PMC7770158

[108] Mccubbrey AL Barthel L Mohning MP Redente EF Mould KJ Thomas SM. Deletion of C-FLIP From CD11b(Hi) Macrophages Prevents Development of Bleomycin-Induced Lung Fibrosis. Am J Respir Cell Mol Biol (2018) 58(1):66–78. doi: 10.1165/rcmb.2017-0154OC 28850249 PMC5941310

[109] Gibbings SL Thomas SM Atif SM McCubbrey AL Desch AN Danhorn T. Three Unique Interstitial Macrophages in the Murine Lung at Steady State. Am J Respir Cell Mol Biol (2017) 57(1):66–76. doi: 10.1165/rcmb.2016-0361OC 28257233 PMC5516280

[110] Chakarov S Lim HY Tan L Lim SY See P Lum J. Two Distinct Interstitial Macrophage Populations Coexist Across Tissues in Specific Subtissular Niches 2019. Science 363(6432):eaau0964. doi: 10.1126/science.aau0964 30872492

[111] Schyns J Bai Q Ruscitti C Radermecker C De Schepper S Chakarov S. Non-Classical Tissue Monocytes and Two Functionally Distinct Populations of Interstitial Macrophages Populate the Mouse Lung. Nat Commun (2019) 10(1):3964. doi: 10.1038/s41467-019-11843-0 31481690 PMC6722135

[112] Tan SY Krasnow MA. Developmental Origin of Lung Macrophage Diversity. Development (2016) 143(8):1318–27. doi: 10.1242/dev.129122 PMC4852511 26952982

[113] Ural BB Yeung ST Damani-Yokota P Devlin JC de Vries M Vera-Licona P. Identification of a Nerve-Associated, Lung-Resident Interstitial Macrophage Subset With Distinct Localization and Immunoregulatory Properties. Sci Immunol (2020) 5(45):eaax8756. doi: 10.1126/sciimmunol.aax8756 32220976 PMC7717505

[114] Keerthivasan S Şenbabaoğlu Y Martinez-Martin N Husain B Verschueren E Wong A. Homeostatic Functions of Monocytes and Interstitial Lung Macrophages Are Regulated *via* Collagen Domain-Binding Receptor LAIR1. Immunity (2021) 54(7):1511–26.e8. doi: 10.1016/j.immuni.2021.06.012 34260887

[115] Zhou B Magana L Hong Z Huang LS Chakraborty S Tsukasaki Y. The Angiocrine Rspondin3 Instructs Interstitial Macrophage Transition *via* Metabolic-Epigenetic Reprogramming and Resolves Inflammatory Injury. Nat Immunol (2020) 21(11):1430–43. doi: 10.1038/s41590-020-0764-8 PMC7815054 32839607

[116] Sabatel C Radermecker C Fievez L Paulissen G Chakarov S Fernandes C. Exposure to Bacterial Cpg DNA Protects From Airway Allergic Inflammation by Expanding Regulatory Lung Interstitial Macrophages. Immunity (2017) 46(3):457–73. doi: 10.1016/j.immuni.2017.02.016 28329706

[117] Bedoret D Wallemacq H Marichal T Desmet C Quesada Calvo F Henry E. Lung Interstitial Macrophages Alter Dendritic Cell Functions to Prevent Airway Allergy in Mice. J Clin Invest (2009) 119(12):3723–38. doi: 10.1172/JCI39717 PMC2786798 19907079

[118] Krljanac B Schubart C Naumann R Wirtz S Culemann S Krönke G. Relmα-Expressing Macrophages Protect Against Fatal Lung Damage and Reduce Parasite Burden During Helminth Infection. Sci Immunol (2019) 4(35). doi: 10.1126/sciimmunol.aau3814 31126996

[119] Meziani L Mondini M Petit B Boissonnas A Thomas de Montpreville V Mercier O. CSF1R Inhibition Prevents Radiation Pulmonary Fibrosis by Depletion of Interstitial Macrophages. Eur Respir J (2018) 51(3):1702120. doi: 10.1183/13993003.02120-2017 29496785

[120] Mccubbrey AL Barthel L Mohning MP Redente EF Mould KJ Thomas SM. Deletion of C-FLIP From CD11b Macrophages Prevents Development of Bleomycin-Induced Lung Fibrosis. Am J Respir Cell Mol Biol (2018) 58(1):66–78. doi: 10.1165/rcmb.2017-0154OC 28850249 PMC5941310

[121] Moore BB Paine R 3rd Christensen PJ Moore TA Sitterding S Ngan R. Protection From Pulmonary Fibrosis in the Absence of CCR2 Signaling. J Immunol (2001) 167(8):4368–77. doi: 10.4049/jimmunol.167.8.4368 11591761

[122] Okuma T Terasaki Y Kaikita K Kobayashi H Kuziel WA Kawasuji M. C-C Chemokine Receptor 2 (CCR2) Deficiency Improves Bleomycin-Induced Pulmonary Fibrosis by Attenuation of Both Macrophage Infiltration and Production of Macrophage-Derived Matrix Metalloproteinases. J Pathol (2004) 204(5):594–604. doi: 10.1002/path.1667 15538737

[123] Ngambenjawong C Gustafson HH Pun SH. Progress in Tumor-Associated Macrophage (TAM)-Targeted Therapeutics. Adv Drug Deliv Rev (2017) 114:206–21. doi: 10.1016/j.addr.2017.04.010 PMC5581987 28449873

[124] Funes SC Rios M Escobar-Vera J Kalergis AM. Implications of Macrophage Polarization in Autoimmunity. Immunology (2018) 154(2):186–95. doi: 10.1111/imm.12910 PMC5980179 29455468

[125] Yang S Yuan HQ Hao YM Ren Z Qu SL Liu LS. Macrophage Polarization in Atherosclerosis. Clin Chim Acta (2020) 501:142–6. doi: 10.1016/j.cca.2019.10.034 31730809

[126] Cassetta L Pollard JW. Targeting Macrophages: Therapeutic Approaches in Cancer. Nat Rev Drug Discov (2018) 17(12):887–904. doi: 10.1038/nrd.2018.169 30361552

[127] Chen J Su FY Das D Das D Srinivasan S Son HN Lee B. Glycan Targeted Polymeric Antibiotic Prodrugs for Alveolar Macrophage Infections. Biomaterials (2019) 195:38–50. doi: 10.1016/j.biomaterials.2018.10.017 30610992 PMC6815516

[128] Su FY Srinivasan S Lee B Chen J Convertine AJ West TE. Macrophage-Targeted Drugamers With Enzyme-Cleavable Linkers Deliver High Intracellular Drug Dosing and Sustained Drug Pharmacokinetics Against Alveolar Pulmonary Infections. J Control Release (2018) 287:1–11. doi: 10.1016/j.jconrel.2018.08.014 30099019 PMC6223132

[129] Bonaventura A Vecchié A Wang TS Lee E Cremer PC Carey B. Targeting GM-CSF in COVID-19 Pneumonia: Rationale and Strategies. Front Immunol (2020) 11:1625. doi: 10.3389/fimmu.2020.01625 32719685 PMC7348297

[130] Merad M Martin JC. Pathological Inflammation in Patients With COVID-19: A Key Role for Monocytes and Macrophages. Nat Rev Immunol (2020) 20(6):355–62. doi: 10.1038/s41577-020-0331-4 PMC7201395 32376901

